# Supplementary material for: Deep interpretable learning of sample representations for characterizing disease states in single-cell transcriptomics
Source: bioRxiv. 2026 Jul 22:2026.07.21.738207. Preprint. [Version 1] doi: 10.64898/2026.07.21.738207 (PMC13419810; doi:10.64898/2026.07.21.738207)
Supplement: Supplementary file 1 [file NIHPP2026.07.21.738207v1-supplement-1.pdf]

bioRxiv preprint doi: https://doi.org/10.64898/2026.07.21.738207; this version posted July 22, 2026. The copyright holder for this preprint (which was not certified by peer review) is the author/funder, who has granted bioRxiv a license to display the preprint in perpetuity. It is made available under a CC-BY 4.0 International license.

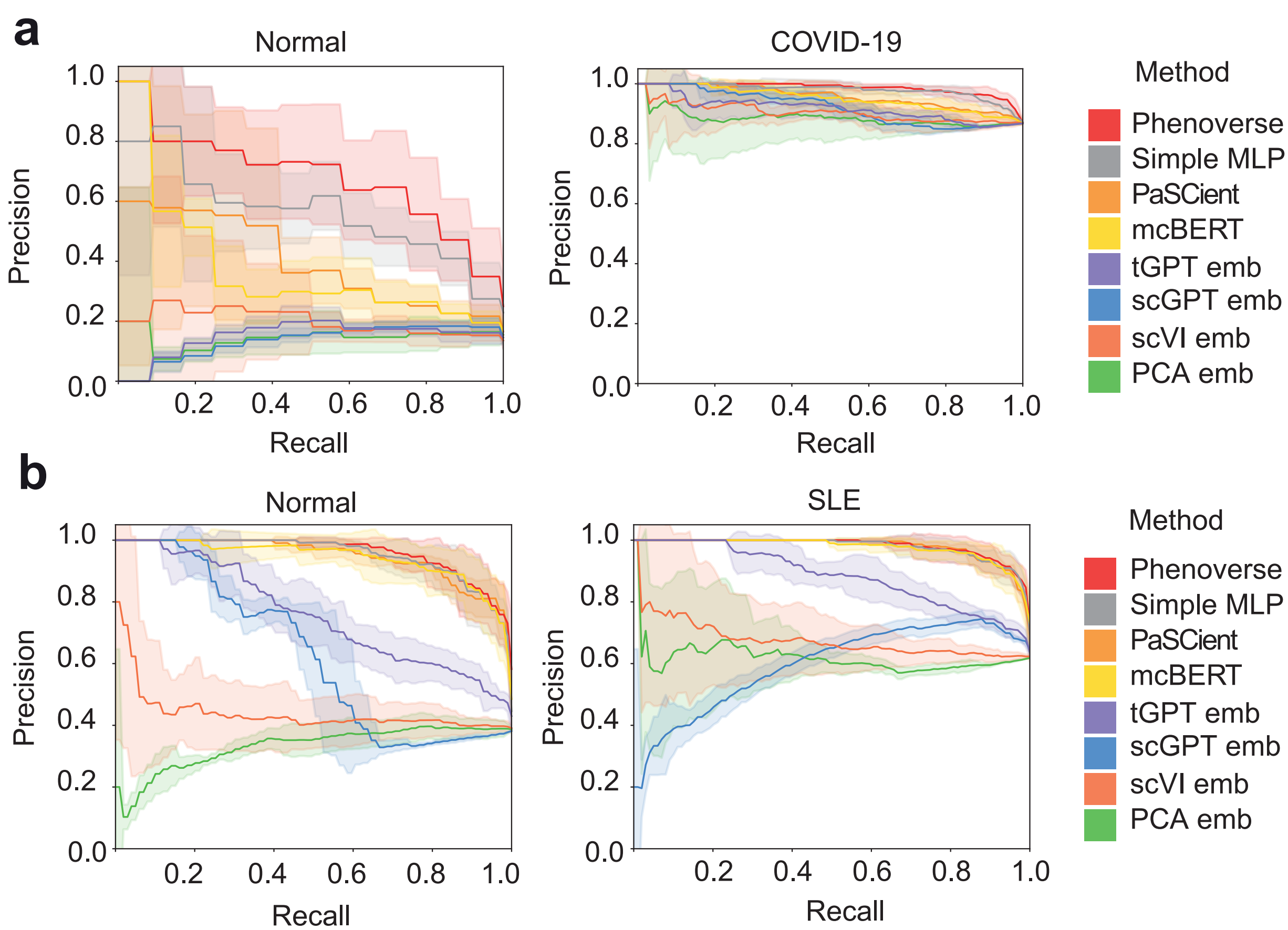

Supplementary Figure 1. Disease state prediction performance of different methods (a) Mean precision-recall curves across five repeated subsampling splits in the COVID-19 dataset, shown separately for the Normal (left) and COVID-19 (right) classes. Shaded bands indicate ±1 standard deviation across splits. (b) Mean precision-recall curves across five repeated subsampling splits in the systemic lupus erythematosus (SLE) dataset, shown separately for the Normal (left) and SLE (right) classes. Shaded bands indicate ±1 standard deviation across splits.

bioRxiv preprint doi: https://doi.org/10.64898/2026.07.21.738207; this version posted July 22, 2026. The copyright holder for this preprint (which was not certified by peer review) is the author/funder, who has granted bioRxiv a license to display the preprint in perpetuity. It is made available under a CC-BY 4.0 International license.

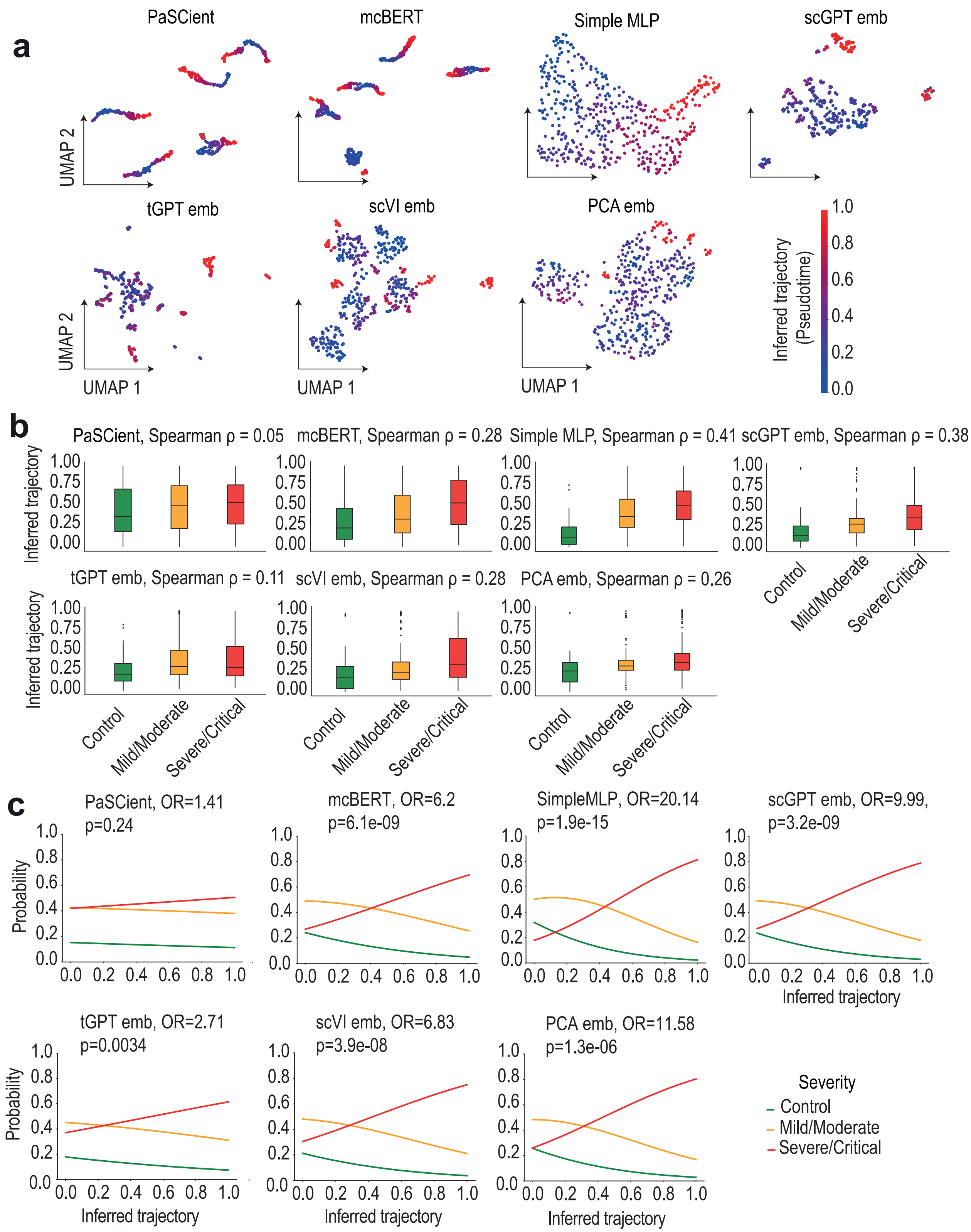

Supplementary Figure 2. Disease trajectory inference performance of baseline methods in the COVID-19 dataset (a) UMAP of sample representations for each baseline method from held-out test samples in the COVID-19 dataset (21), colored by inferred trajectory (pseudotime). (b) Box plots showing the distribution of inferred trajectories across clinical severity categories (control, mild/moderate, and severe/critical) for each baseline method. Box plots show the median (center line), interquartile range (IQR) box bounds: 25th–75th percentile, 1.5×IQR (whiskers), and points outside 1.5×IQR as outliers. (c) Line plots showing ordinal logistic regression predicted probabilities for each clinical severity category along the inferred trajectory for different baseline methods. Odds ratios (OR) and associated p-values are indicated in each panel title.

bioRxiv preprint doi: https://doi.org/10.64898/2026.07.21.738207; this version posted July 22, 2026. The copyright holder for this preprint (which was not certified by peer review) is the author/funder, who has granted bioRxiv a license to display the preprint in perpetuity. It is made available under a CC-BY 4.0 International license.

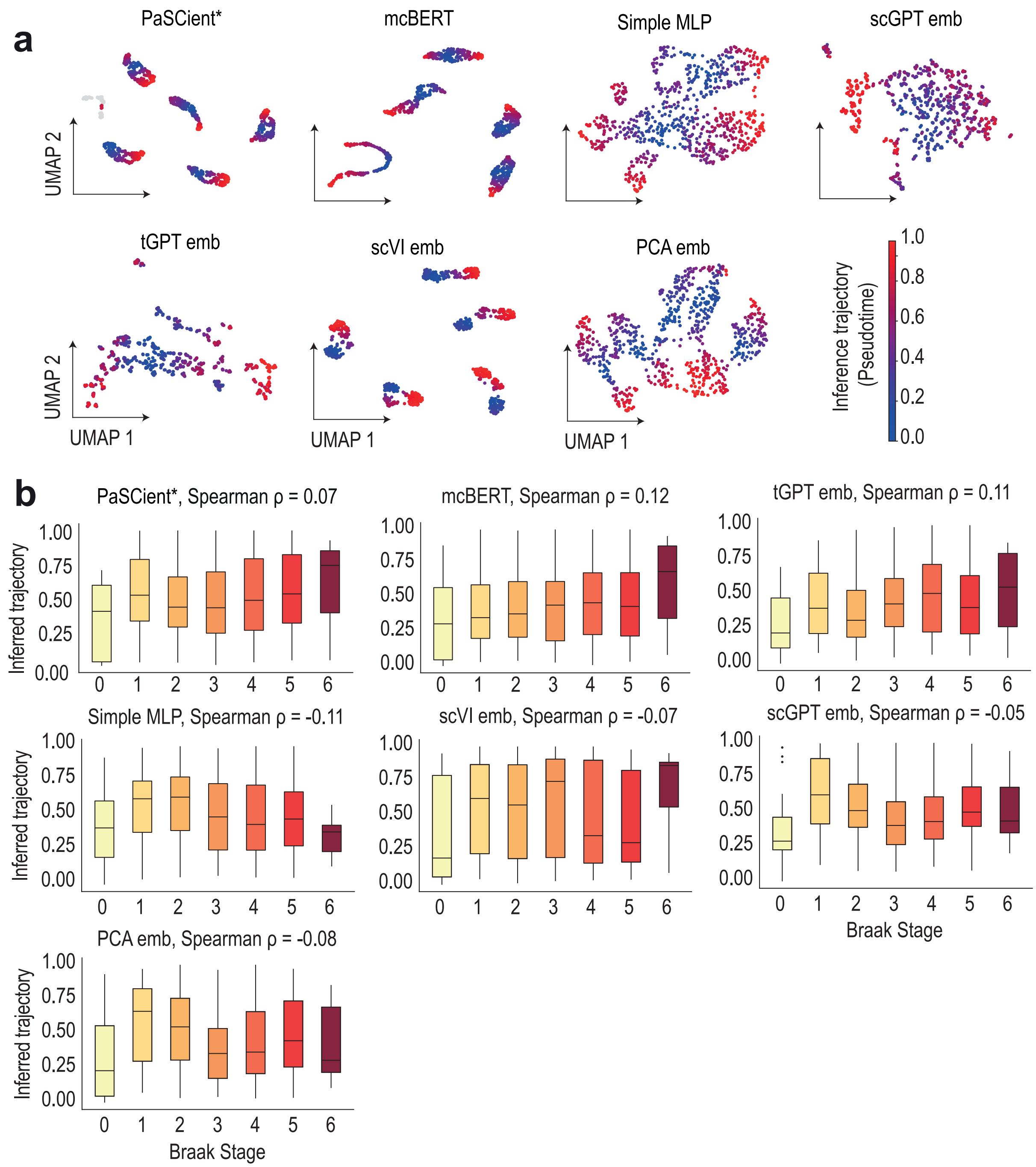

Supplementary Figure 3. Correlation of inferred trajectories of baseline methods with Braak staging in Alzheimer's disease (a) UMAP of sample representations for each baseline method from held-out test samples (26), colored by inferred trajectory (pseudotime). * indicates that pseudotime values were undefined for a subset of held-out samples. (b) Box plots showing the distribution of inferred trajectories across Braak stages (0–6) for each baseline method. Box plots show the median (center line), interquartile range (IQR) box bounds: 25th–75th percentile, 1.5×IQR (whiskers), and points outside 1.5×IQR as outliers. * indicates that pseudotime values were undefined for a subset of held-out samples.

bioRxiv preprint doi: https://doi.org/10.64898/2026.07.21.738207; this version posted July 22, 2026. The copyright holder for this preprint (which was not certified by peer review) is the author/funder, who has granted bioRxiv a license to display the preprint in perpetuity. It is made available under a CC-BY 4.0 International license.

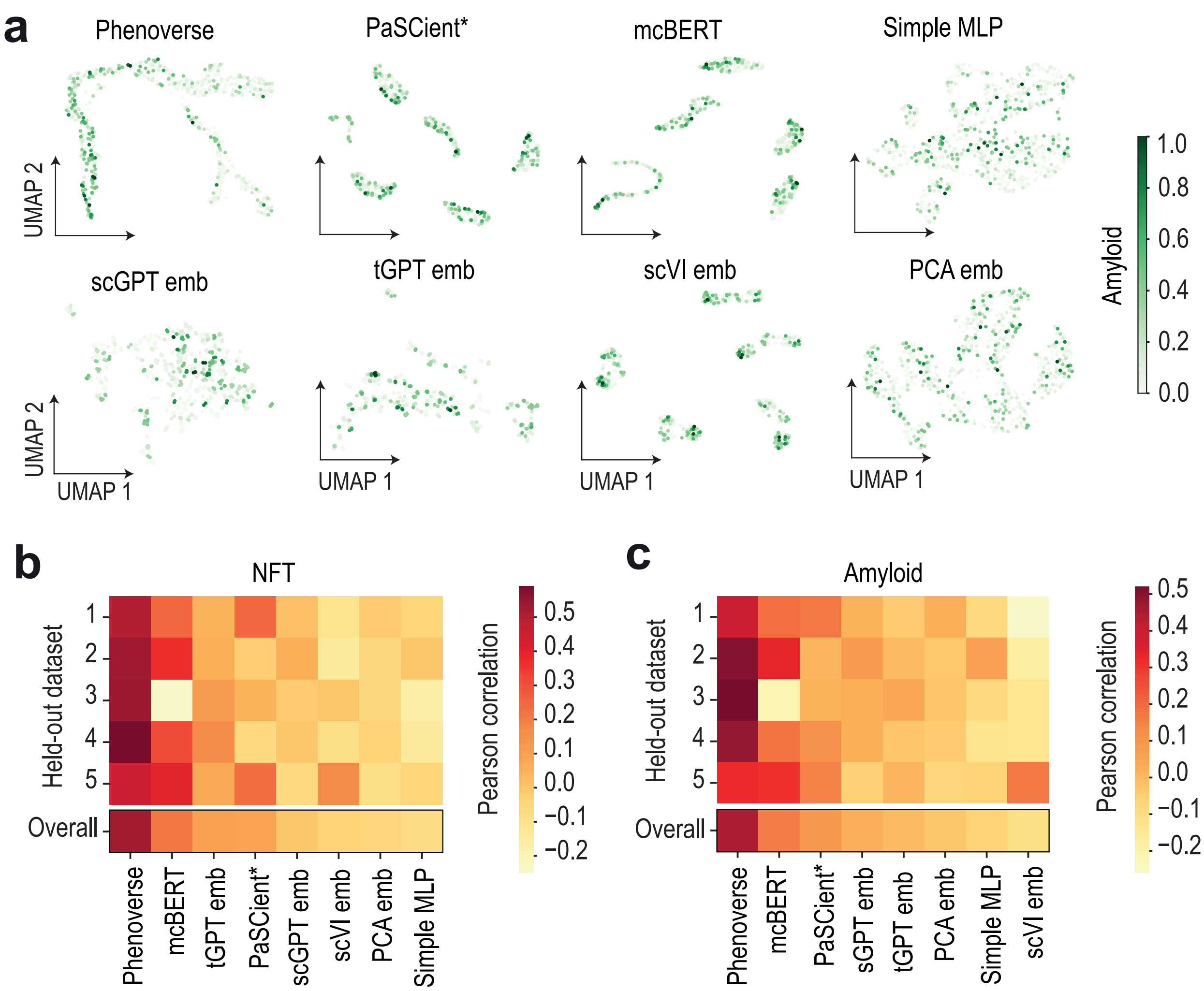

Supplementary Figure 4. Correlation of inferred trajectories of baseline methods with neuropathological burden in Alzheimer's disease (a) UMAPs of sample representations from held-out test samples in the Alzheimer's disease dataset (26), colored by amyloid burden. * indicates that pseudotime values were undefined for a subset of held-out samples. (b-c) Heatmaps showing the Pearson correlation between inferred trajectory and NFT burden (b) and amyloid burden (c) across five repeated subsampling validation splits. * indicates that pseudotime values were undefined for a subset of held-out samples.

bioRxiv preprint doi: https://doi.org/10.64898/2026.07.21.738207; this version posted July 22, 2026. The copyright holder for this preprint (which was not certified by peer review) is the author/funder, who has granted bioRxiv a license to display the preprint in perpetuity. It is made available under a CC-BY 4.0 International license.

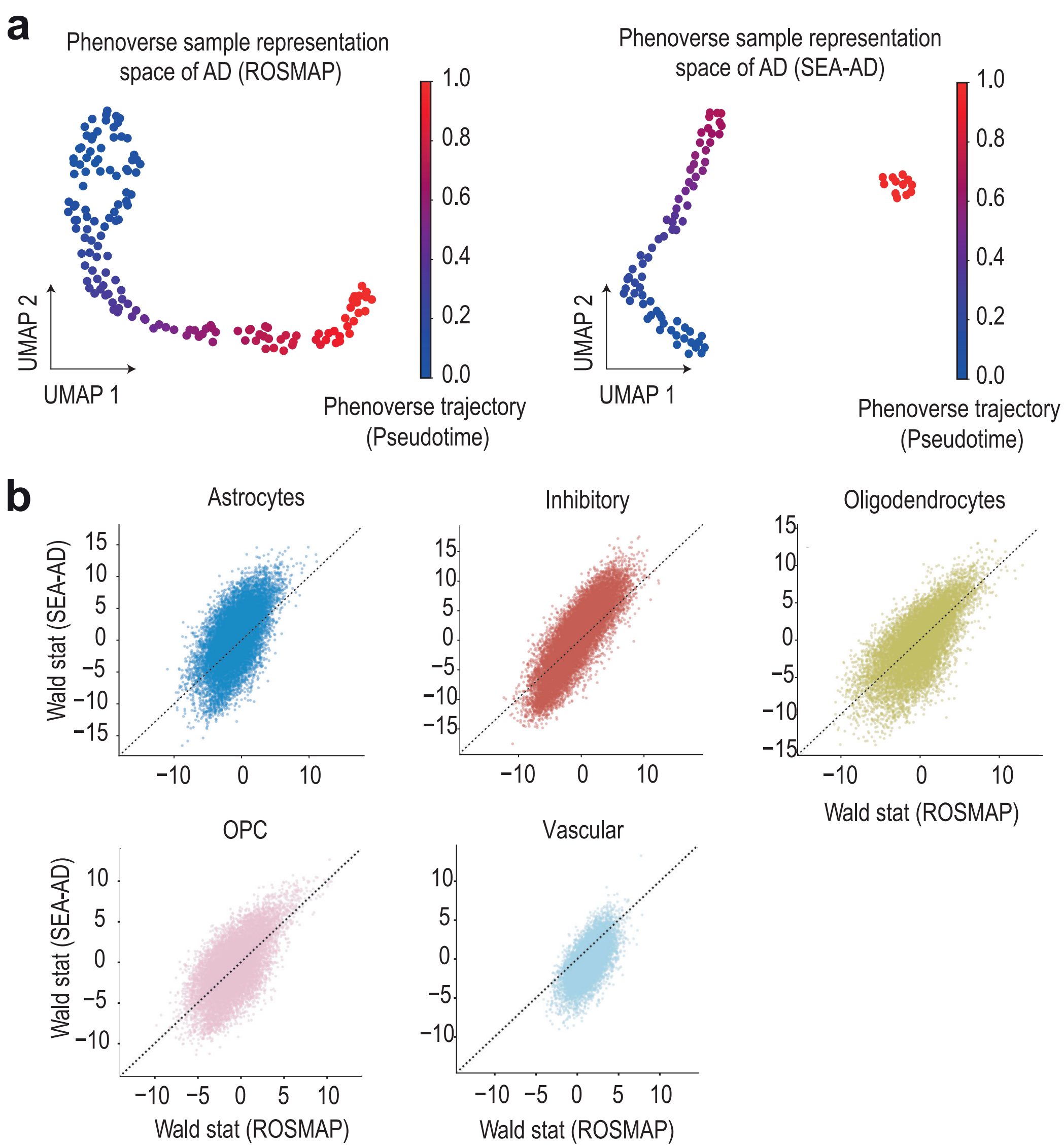

Supplementary Figure 5. Cross-cohort trajectory inference and gene-level reproducibility in Alzheimer's disease (a) UMAPs of Phenoverse sample representations from held-out ROSMAP (left) and SEA-AD (right) samples, colored by inferred trajectory (pseudotime). (b) Scatter plots showing the cross-cohort reproducibility of gene-level Wald statistics from Phenoverse trajectory-based differential expression analysis across different cell types.

bioRxiv preprint doi: https://doi.org/10.64898/2026.07.21.738207; this version posted July 22, 2026. The copyright holder for this preprint (which was not certified by peer review) is the author/funder, who has granted bioRxiv a license to display the preprint in perpetuity. It is made available under a CC-BY 4.0 International license.

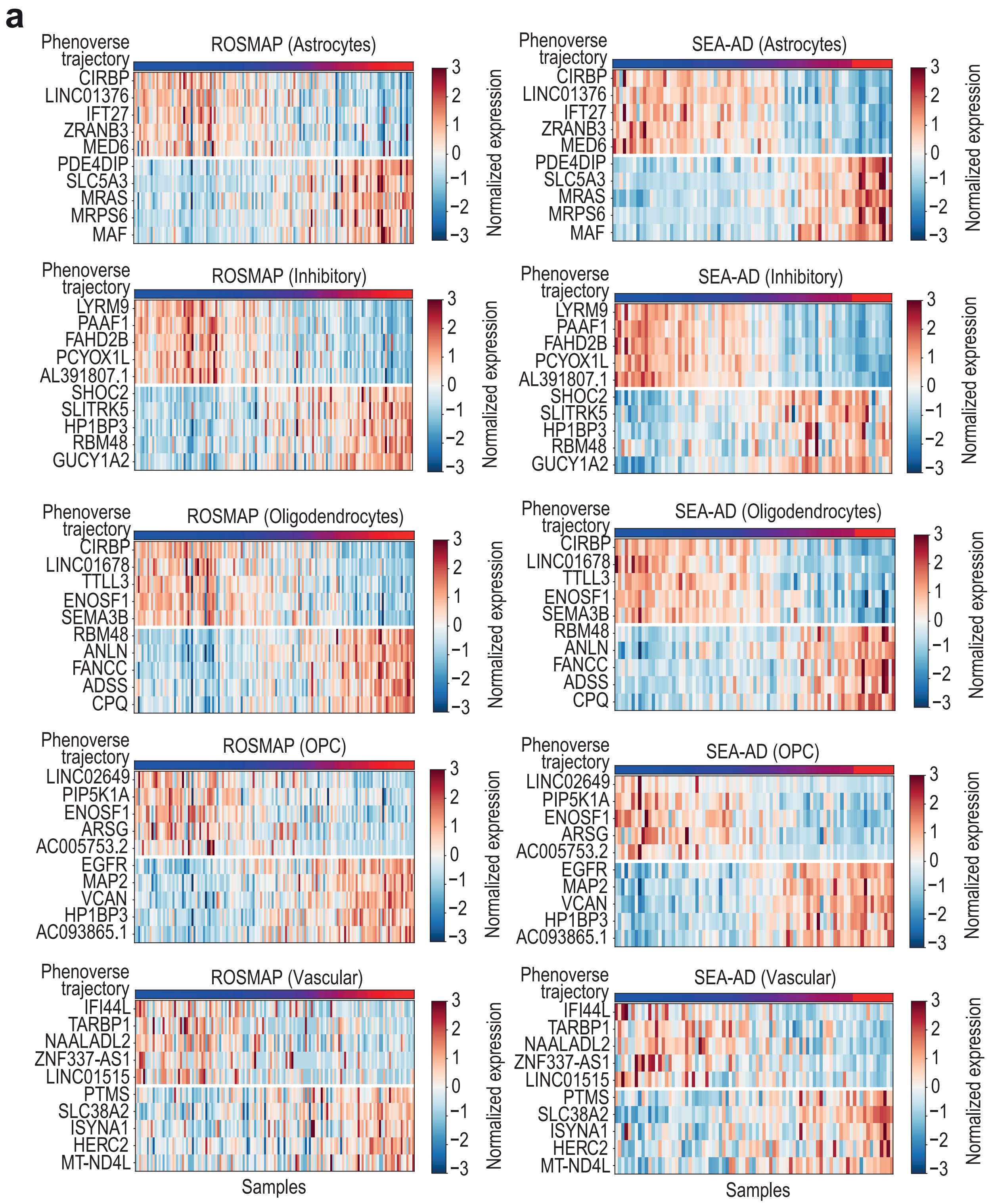

Supplementary Figure 6. Expression patterns of Phenoverse trajectory-associated genes (a) Heatmaps showing expression patterns of top upregulated and downregulated trajectory-associated genes across different cell types. Genes were selected based on Wald statistics from the ROSMAP cohort. Expression values are shown as row-normalized Z-scores for ROSMAP (left) and SEA-AD (right) samples. Rows correspond to genes, and columns correspond to samples ordered by increasing pseudotime.

bioRxiv preprint doi: https://doi.org/10.64898/2026.07.21.738207; this version posted July 22, 2026. The copyright holder for this preprint (which was not certified by peer review) is the author/funder, who has granted bioRxiv a license to display the preprint in perpetuity. It is made available under a CC-BY 4.0 International license.

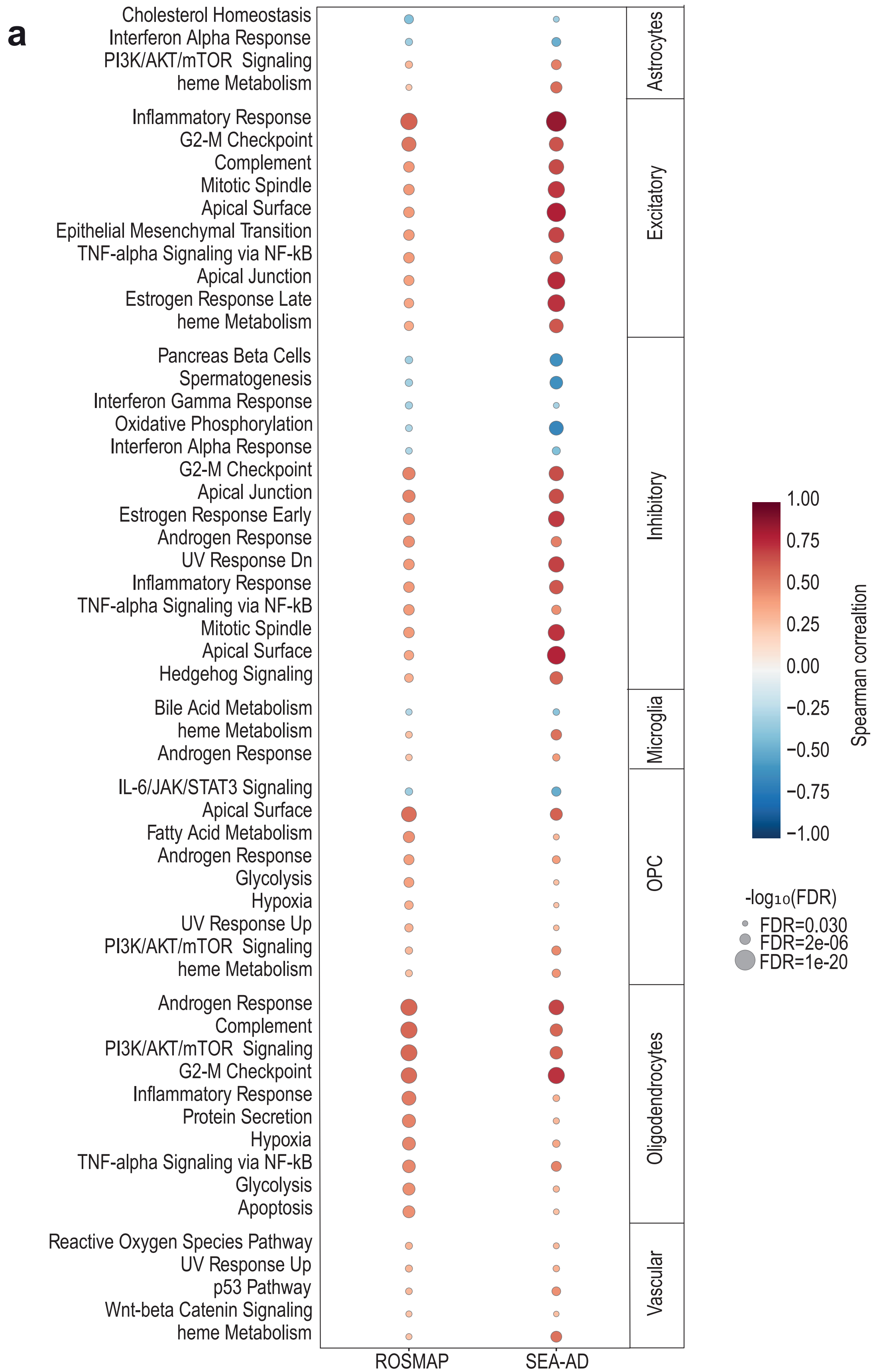

Supplementary Figure 7. Molecular programs along Phenoverse-inferred trajectories in Alzheimer's disease (a) Dot plot showing the top 10 significant hallmark pathways along Phenoverse-inferred trajectories across seven cell types in ROSMAP and SEA-AD cohorts. Dot size represents the magnitude of the Spearman correlation between pathway activity scores and the Phenoverse-inferred trajectory. Color indicates direction relative to the inferred trajectory (red: enriched; blue: depleted).

bioRxiv preprint doi: https://doi.org/10.64898/2026.07.21.738207; this version posted July 22, 2026. The copyright holder for this preprint (which was not certified by peer review) is the author/funder, who has granted bioRxiv a license to display the preprint in perpetuity. It is made available under a CC-BY 4.0 International license.

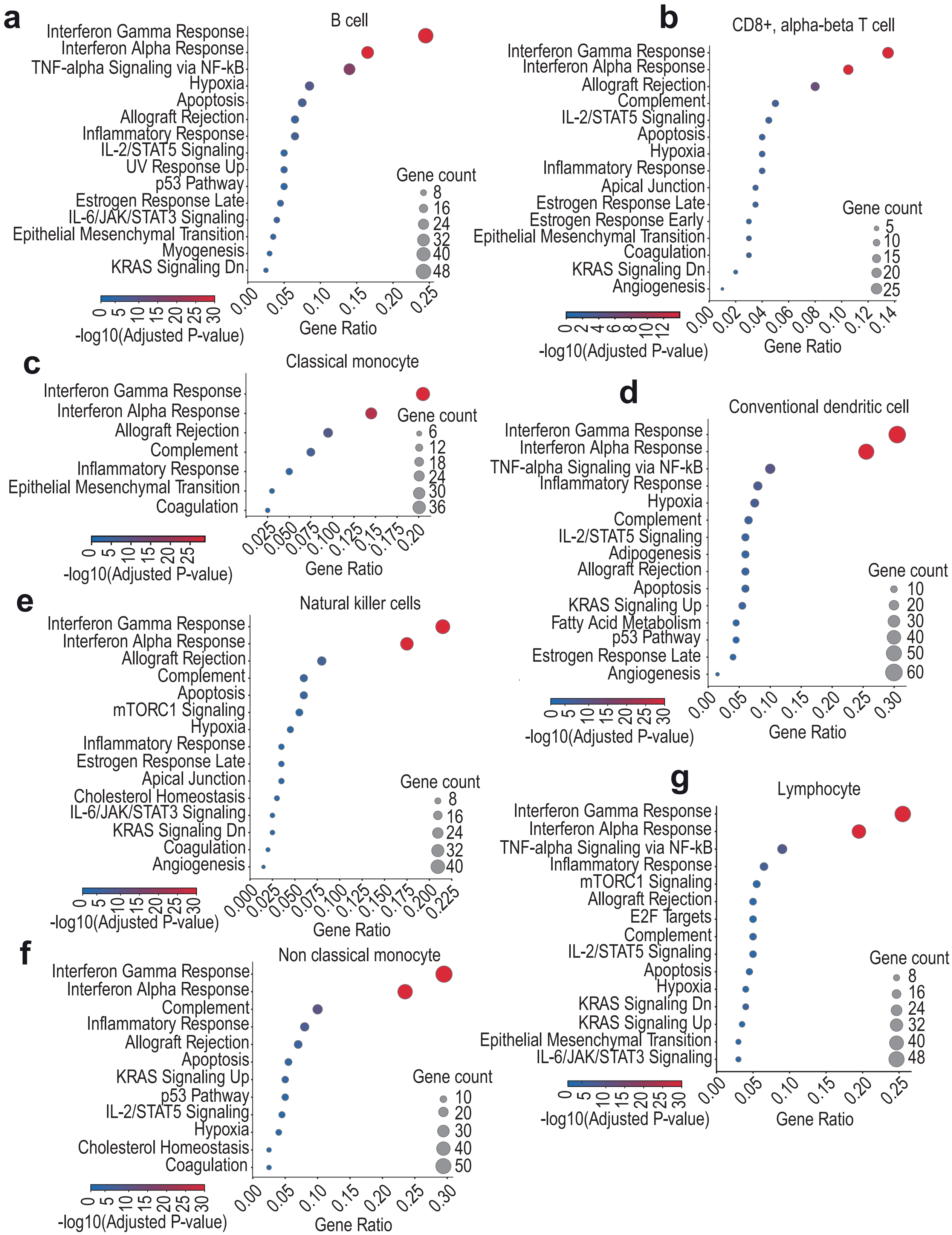

Supplementary Figure 8. Prototype enriched programs in systemic lupus erythematosus (a-g) Dot plots illustrating enrichment of top prototype 3 gene programs in B cell (a), CD8+ alpha-beta T cell (b), classical monocyte (c), conventional dendritic cell (d), natural killer cell (e), non-classical monocyte (f), and lymphocyte (g).
